# Protective effects of medicinal honey against doxorubicin-induced hepatorenal toxicity in rats: A novel grading index (PAD score) based on phenolic content and antioxidant capacity

**DOI:** 10.1016/j.metop.2025.100440

**Published:** 2026-01-31

**Authors:** Mahdi Honarbakhsh, Nafiseh Erfanian, Amir Hossein Saberi, Pouria Mohammad Parast Tabas, Motahhareh Mohammadi, Ahmad Bavali-Gazik, Asghar Zarban

**Affiliations:** a Student Research Committee, Birjand University of Medical Sciences, Birjand, Iran; b Assistant Professor of Molecular Medicine, Cellular and Molecular Research Center, Birjand University of Medical Sciences, Birjand, Iran; c Student Research Committee, Iran University of Medical Sciences, Tehran, Iran; d Professor of Biochemistry, Clinical Biochemistry Department, Faculty of Medicine, Birjand University of Medical Sciences, Birjand, Iran

**Keywords:** Doxorubicin, Honey, PAD score, Hepatoprotection, Nephroprotection, Antioxidants

## Abstract

**Background:**

Doxorubicin is an effective anticancer drug whose clinical use is limited by oxidative liver and kidney toxicity. High-PAD-score honey, a novel index reflecting honey quality and bioactive potential, is rich in phenolic compounds and antioxidants and may mitigate these toxic effects. This study aimed to evaluate the protective effects of PAD-classified honey against doxorubicin-induced hepatorenal toxicity in rats.

**Methods:**

Fifteen honey samples collected from different regions of Iran were biochemically characterized using a novel PAD scoring system calculated as the sum of four parameters: total phenolic content, antioxidant capacity, protein concentration, and diastase activity. Based on their PAD values, honeys were classified into high-, moderate-, and low-PAD categories and pooled accordingly. Thirty-five male Wistar rats were randomly assigned to five groups and orally treated with 20% (w/v) PAD-classified honey for four weeks, followed by doxorubicin administration (20 mg/kg, i.p.) to induce hepatorenal toxicity. Serum biochemical markers, oxidative stress indices, and histopathological alterations in liver and kidney tissues were subsequently evaluated.

**Results:**

Doxorubicin increased liver and kidney injury markers, oxidative stress parameters, and glucose levels. Medium- and high-PAD honeys improved biochemical homeostasis, enhanced antioxidant defense, and, as confirmed by histopathological analysis, attenuated hepatic and renal degeneration, necrosis, inflammation, and structural damage.

**Conclusion:**

High-PAD-score honey, owing to its antioxidant and phenolic properties, may exert protective effects against doxorubicin-induced organ toxicity in experimental models. However, further mechanistic studies and carefully designed investigations are required before any implications for supportive or clinical use can be considered.

## 1 Introduction

Doxorubicin (DOX) is one of the most widely used anthracycline chemotherapeutic agents and plays a central role in the treatment of various malignancies, including breast, ovarian, and lung cancers, soft tissue sarcomas, Hodgkin and non-Hodgkin lymphomas, and acute leukemias. Its cytotoxic activity primarily results from inhibition of topoisomerase II, leading to DNA damage, cell cycle arrest, and apoptotic cell death [1].

The hepatotoxic effects of doxorubicin largely result from excessive oxidative stress, accompanied by a marked reduction in aromatic amino acids such as phenylalanine and tyrosine, as well as disturbances in hepatic enzyme function. These biochemical alterations are reflected by elevated serum aminotransferase levels, which serve as indicators of liver injury and dysfunction [[2], [3], [4], [5]]. In renal tissues, doxorubicin induces oxidative stress and apoptosis, thereby contributing to the development of acute kidney injury (AKI) and chronic kidney disease (CKD) [6]. Clinical complications such as proteinuria and hypertension further exacerbate patient morbidity and adversely affect quality of life [7].

Honey is a natural product rich in phenolic compounds, which exhibit strong radical-scavenging activity and substantially enhance total antioxidant capacity [8,9]. Experimental studies have shown that honey consumption can upregulate key antioxidant enzymes, such as catalase and glutathione peroxidase, both of which play pivotal roles in detoxifying reactive oxygen species [10,11]. Honey also reduces lipid peroxidation, thereby protecting cells from oxidative injury [12,13].

In addition, its natural sugars primarily fructose and glucose provide metabolic support for hepatocytes and facilitate detoxification processes [14,15]. Evidence also suggests that honey may reduce the risk of kidney stone formation by modulating urinary pH and inhibiting crystal deposition [[16], [17], [18]].

However, the biochemical composition and therapeutic potential of honey vary substantially depending on floral origin [19]. While several classification approaches such as color categories and physicochemical profiling have been used [[20], [21], [22]], These systems do not adequately reflect the bioactive and medicinal value of honey. To address this limitation, a newly developed biochemical index, known as the PAD score, evaluates honey quality based on four functional attributes: phenolic composition, total protein content, antioxidant capacity, and diastase enzymatic activity. Together, these parameters represent the antioxidant strength, biochemical richness, and enzymatic integrity of honey, enabling standardized identification of samples with superior bioactive potential. The PAD system categorizes honey into six quality grades according to phenolic and protein content, antioxidant capacity, and diastase activity (24, 25). Although the PAD score has been introduced as a standardized tool for grading honey quality, no experimental study has yet examined whether stratifying honeys according to this index translates into differential protection against chemotherapeutic organ injury. In the present study, low-, medium-, and high-PAD-score honeys (LPH, MPH, and HPH, respectively) were used to evaluate whether honey with higher antioxidant and cytoprotective properties can attenuate doxorubicin-induced liver and kidney toxicity.

## 2 Materials and methods

### 2.1 Preparation of honey samples and quality assessment

Fifteen honey samples were collected from various regions of Iran between 2023 and 2024. The quality of each honey sample was evaluated using the PAD scoring system, an integrated biochemical index incorporating four major parameters: total phenolic content, antioxidant capacity, protein concentration, and diastase enzymatic activity. Each parameter contributed equally to the final PAD score to ensure a balanced assessment of the bioactive and nutritional properties of honey. Total phenolic content, antioxidant capacity, and diastase enzyme activity were quantified using the ZANTOX DPPH assay kit (ZANTOX, Iran) according to the manufacturer's instructions. Protein concentration was determined separately using a Bradford assay kit (KalaZist, Iran). All measured values were normalized and incorporated into the PAD scoring formula to generate a standardized biochemical profile for each honey sample.

The PAD score was calculated as an unweighted composite index derived from the summed normalized values of total phenolic content, antioxidant capacity, protein concentration, and diastase activity Table 2

Based on their PAD values, honey samples were categorized into three groups: high-PAD-score honey (HPH), moderate-PAD-score honey (MPH), and low-PAD-score honey (LPH). Five samples from each category were pooled to generate representative composite samples for subsequent experimental evaluations. For experimental use, a stock solution was prepared by dissolving 1 g of honey in 1 mL of sterile distilled water to obtain a 100% (w/v) solution. The mixture was then filtered through a 0.45 μm syringe filter to remove impurities, and further dilutions were prepared as required for downstream analyse.

All assays used to construct the PAD score were selected based on widely accepted, up-to-date approaches for honey quality and bioactivity assessment and were conducted according to established methods and the corresponding literature. In addition, the dosing regimen used in the in vivo experiment (honey concentration and doxorubicin administration protocol) was chosen based on previously published studies in comparable experimental models [23].

### 2.2 Experimental animals

Animals were grouped into five cages and maintained at the IR.BUMS.REC University of Medical Sciences Laboratory Research Center under controlled conditions of a natural light/dark cycle and a temperature of 22 ± 2 °C. Throughout the intervention, the animals were fed with nutrient pellets and tap water. Additionally, the ethical approval for this research was granted by the Research Ethics Committee of University of Medical Sciences (Ethical Code: IR.BUMS.REC). Furthermore, the experiments were conducted in accordance with the principles outlined in the Guide for the Care and Use of Laboratory Animals, prepared by the National Academy of Sciences and published by the National Institutes of Health.

### 2.3 Treatment

Thirty-five male Wistar rats were randomly assigned to five groups (Table 1); following Randomization, the rats were enrolled in the study and received daily treatments according to their group labels. Consistent with this protocol, the control groups, including negative and positive controls, received 1 mL of water, while the treatment groups, comprising HPH, MPH and LPH, received a freshly prepared 20% (w/v) aqueous honey solution as a single dose. This regimen was continued for four weeks. At the end of the treatment period, the positive control and all three honey-treated groups were subjected to toxic induction using DOX (20 mg/kg, Pfizer) intraperitoneally for three consecutive days (days 31, 32, and 33). Forty-eight hours post-induction, the rats were anesthetized with an intraperitoneal injection of ketamine hydrochloride (65 mg/kg) and xylazine (10 mg/kg), and subsequently euthanized. Blood samples were collected via cardiac puncture and the inferior vena cava, and the serum fraction was extracted for biochemical and antioxidant measurements. Additionally, liver and kidney tissues were sampled for histopathological examinations.

**Table 1:** Treatment groups of the study.

Group	Classification	Group Number
Control	control	1
DOX-induced toxicity	Independent of honey intervention (DOX)	2
Treated and DOX-induced toxicity	LPH	3
	MPH	4
	HPH	5

**Abbreviations:** DOX: doxorubicin; PAD: Phenolic Content and Protein, Antioxidant Capacity and Diastase Activity; LPH: low PAD score honey; MPH: medium PAD score honey; HPH: high PAD score honey.

Serum tubes and tissue samples were labelled with anonymized codes so that the pathologist performing histopathological scoring and the laboratory personnel conducting biochemical and antioxidant assays were blinded to group allocation.

### 2.4 Biochemical assays

Serum levels of fasting blood glucose (FBS), liver function markers including alanine transaminase [24], aspartate transaminase (AST), alkaline phosphatase [25], cardiac damage markers including lactate dehydrogenase (LDH), creatine phosphokinase (CPK), cholesterol (Chol), triglycerides (TG), low-density lipoprotein (LDL), and high-density lipoprotein (HDL), and renal function markers including albumin [26], uric acid (URIC ACID), creatinine (Cr), and urea were assessed using a biochemical autoanalyzer (Prestige-i24, Japan) and commercial diagnostic kits (Pars Azmoon Co.)**.**

### 2.5 Effects of PAD-scored honey on oxidative stress and lipid peroxidation markers

The total phenolic content of honey samples was measured using the Folin–Ciocalteu reagent (FCR) method, honey protein concentration was determined by the Bradford assay, and diastase enzyme activity was assessed using an enzymatic method. The antioxidant capacity of honey and serum samples was evaluated using the DPPH assay, along with ferric reducing antioxidant power (FRAP) and total thiol content assessed by the Benzie and Strain and Ellman methods, respectively. In contrast, malondialdehyde (MDA) levels were measured using the TBARS assay as an index of lipid peroxidation and oxidative damage. All assays were performed using commercial kits (Kavosh Arian Azma Company, Iran) according to the manufacturer's instructions [[27], [28], [29], [30], [31], [32], [33], [34], [35], [36]].

### 2.6 Histopathological examinations

Liver and kidney tissues were dissected and fixed in 10% neutral buffered formalin for at least 48 h, followed by routine tissue processing. Finally, prepared tissue sections were cut into 5 μm slices using a Shandon Citadel 315 microtome (UK) and stained with hematoxylin and eosin (H&E). The sections were then observed using a light microscope (Olympus, Japan) for histopathological changes. The severity of histopathological changes was assessed using the histopathological activity index derived from the Kondoll and Banff index [37,38] by a single experienced who was blinded to group allocation.

### 2.7 Statistical analyses

Statistical analysis was performed using GraphPad Prism 10 software. The shapiro-wilk test, with a significance level of *p* > 0.05, was used to determine the normal distribution of data. For normally distributed data, one-way analysis of variance (ANOVA) followed by Tukey's multiple comparison test was used to compare differences between group. In non-parametric conditions, the Kruskal-Wallis test followed by the Mann-Whitney *U* test was used to examine significant differences between groups. Significance levels of *p* < 0.001, 0.05, and 0.01 were checked and considered. Results are expressed as (mean ± SE and mean ± SD). A post hoc power analysis was conducted using G∗Power software (version 3.1) for a one-way ANOVA design with five groups (n = 7 rats per group, total n = 35) and a significance level of α = 0.05. The analysis indicated that the present sample size provides adequate statistical power (approximately 0.75) to detect large effect sizes (f = 0.6), while smaller or moderate effects may not have remained detectable.

## 3 Results

### 3.1 Honey classification based on PAD score

Analysis of the honey samples revealed clear differences in biochemical composition according to PAD scores. The high-PAD-score honey (HPH) group (samples 1–5; PAD score >400) exhibited the highest antioxidant activity (DPPH), total phenolic content, and protein concentration, along with relatively higher diastase activity and color intensity. The moderate-PAD-score honey (MPH) group (samples 6–10; PAD score = 200–400) showed intermediate levels of antioxidant activity, phenolic compounds, and protein content, which were lower than those observed in the HPH group but higher than those in the low-PAD-score honey (LPH) group. In contrast, the LPH group (samples 11–15; PAD score <200) exhibited the lowest levels of antioxidant activity, phenolic compounds, and protein concentration, as well as the lowest diastase activity and color intensity (Table 2).

**Table 2:** Comparison between honey classifications in 15 types of honey samples (5 samples/group) based on PAD Score.

Honey Sample	Antioxidant (DPPH) mg TE/100 g	Phenolic content (FCR) GAE/100 g	Proteins mg	Diastase activity IU/ML	Color	PAD Score^a^	Classification of honeys^b^
1	207.4	164.1	246	19.5	3.1445	637	HPH
2	207.8	132.3	238	19.05	1.232	597.15	
3	187.2	118	228	18.9	1.7	552.1	
4	170	178	340.9	24.6	1.199	613.5	
5	272.9	136	250	30.75	1.5	689.65	
6	87.5	87.1	113.2	24.3	0.45	312.1	MPH
7	96.1	74	114.1	23.7	0.283	307.9	
8	87.6	66.8	123.3	25.8	0.535	303.5	
9	61.5	66.2	144	26.7	0.554	298.4	
10	61.3	94	114.9	14.1	0.478	284.3	
11	11	16.5	78.5	15.3	0.26	121.3	LPH
12	5	19.3	74.6	6.9	0.198	118.8	
13	5.6	19	81.4	12.6	0.16	118.6	
14	6	12.1	89.7	7.2	0.154	109.8	
15	16.3	20.1	58.6	12.9	0.251	107.9	

**Abbreviations:** DOX: doxorubicin; LPH: low PAD score honey; MPH: medium PAD score honey; HPH: high PAD score honey.

a Determination of the PAD Index by specified factors for honey quality assessment.

b The five honey samples in each group were mixed and used as a standard honey for treating the rats based on PAD Index.

### 3.2 Differences between groups based on PAD index

**Fig. 1:**
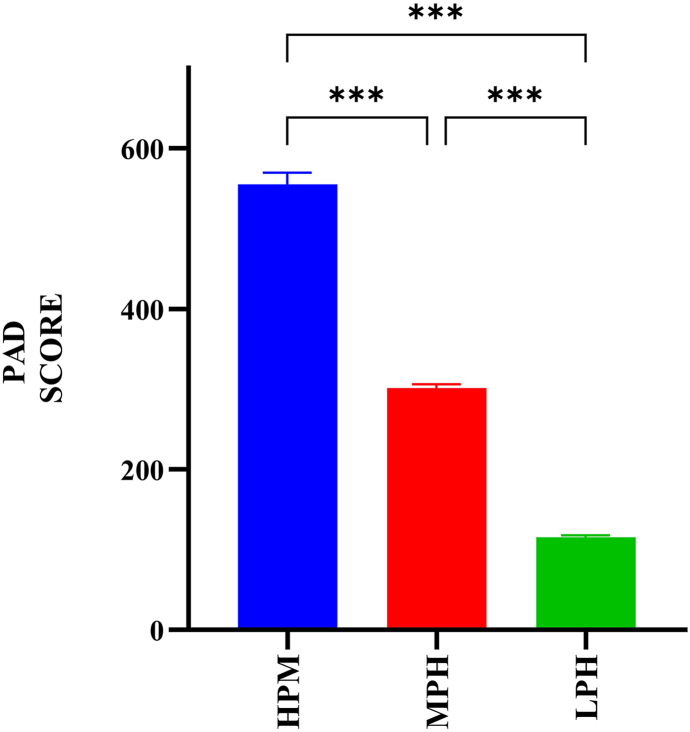
Comparison of honey samples based on PAD score. PAD scoring was determined using phenolic compounds, antioxidant capacity, protein content, and diastase enzyme activity. All values are presented as mean ± SEM. *p* < 0.001 indicates statistically significant differences.

Comparisons between HPH, MPH, and LPH were all statistically significant (*p* < 0.001). This indicates a substantial difference in mean PAD scores among the groups, confirming that the honey quality levels are clearly distinct (Fig. 1).

### 3.3 Biochemistry

#### 3.3.1 Renal function markers

**Fig. 2:**
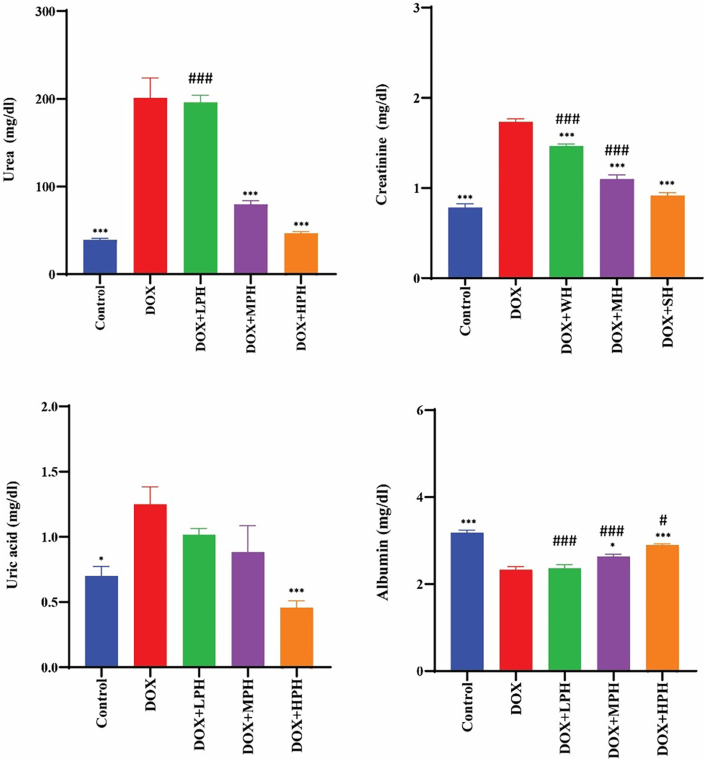
Protective role of graded honey against DOX-Induced nephrotoxicity. All data are presented as mean ± SE (mg/dL). ^#^Compare with Control, ∗ Compare with DOX, Statistical significance is indicated as: ^#^, ∗*p* < 0.05; ^##^, ∗∗<*p* < 0.01; ^###^, ∗∗∗*p* < 0.001.

Doxorubicin administration significantly increased serum urea, creatinine, and uric acid levels compared with the control group (p < 0.001), indicating renal toxicity. Treatment with medium- and high-PAD-score honeys (MPH and HPH) significantly reduced urea and creatinine levels compared with the DOX group (p < 0.001), whereas low-PAD-score honey (LPH) showed no significant effect. Similarly, uric acid levels were markedly reduced in the HPH group, even below control values (p < 0.001), with HPH demonstrating greater efficacy than MPH (p < 0.05). Albumin levels were partially restored following MPH and HPH treatment (p < 0.05), with HPH exhibiting the most pronounced effect, although values remained lower than those observed in the control group (Fig. 2).

**Table 3:** Antioxidant parameters in serum samples among study groups. Values are presented as mean ± SEM (μmol/L). Statistical annotations.

Classification of honeys	Groups	FRAP, mean ± SEM (μmol/L)	MDA, mean ± SEM (μmol/L)	DPPH, mean ± SEM (μmol/L)	Thiol, mean ± SEM (μmol/L)
Independent of honey intervention	Control	317 ± 13.2	0.17 ± 0.0153	384 ± 22	170 ± 9.58
Independent of honey intervention	DOX	244 ± 10.9 a, ∗	0.317 ± 0.0109 a, ∗∗∗	304 ± 8.74 a, ∗	116 ± 11.2 a, ∗
LPH	DOX + LPH	310 ± 21.6 b, ∗	0.307 ± 0.0136 a, ∗∗∗	310 ± 21.6 a, ∗	119 ± 6.12 a, ∗
MPH	DOX + MPH	326 ± 17.2 b, ∗∗	0.262 ± 0.0229 a, ∗∗	326 ± 17.2	128 ± 4.52
HPH	DOX + HPH	356 ± 13.9 b, ∗∗∗	0.247 ± 0.0194 a, ∗	321 ± 11.6	158 ± 15.2 b, ∗

a: significant difference compared to the Control group, b: significant difference compared to the DOX group. Level of significance: ∗*p* < 0.05; ∗∗*p* < 0.01; ∗∗∗*p* < 0.001. Parameters include: FRAP (ferric reducing antioxidant power), MDA (malondialdehyde), DPPH radical scavenging activity, and total Thiol content. **Abbreviation:** DOX: doxorubicin; LPH: low PAD score honey; MPH: medium PAD score honey; HPH: high PAD score honey.

#### 3.3.2 Liver enzymes

**Fig. 3:**
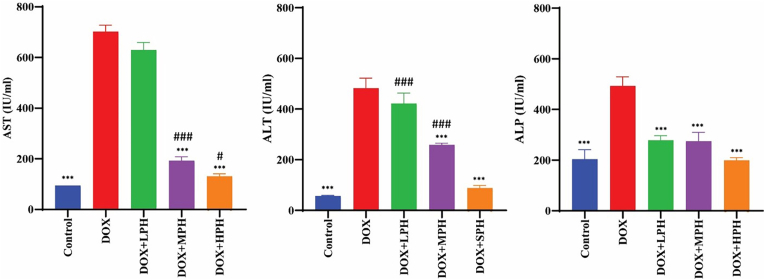
Liver function markers among study groups. Levels of ALT, AST, and ALP are shown. All data are presented as mean ± SEM (IU/mL). ^#^Compare with Control, ∗ Compare with DOX, Statistical significance is indicated as: ^#^, ∗*p* < 0.05; ^##^, ∗∗*p* < 0.01; ^###^, ∗∗∗*p* < 0.001.

DOX induced significant elevations in ALT, AST, and ALP levels (*p* < 0.001). All honey treatments reduced these enzyme levels, with MPH and HPH restoring them to near control values (*p* < 0.001). LPH produced a moderate reduction, which was statistically significant but less pronounced than MPH and HPH (Fig. 3).

#### 3.3.3 Cardiac enzymes

**Fig. 4:**
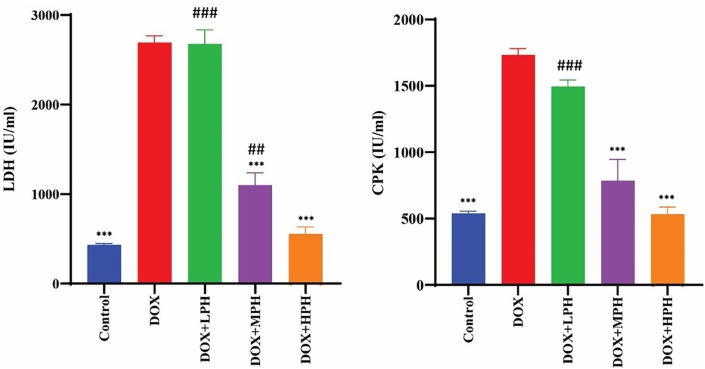
Cardioprotective Effect of Honey Against DOX-Induced Myocardial Damage. All data are presented as mean ± SEM (mg/dL). ^#^Compare with Control, ∗ Compare with DOX, Statistical significance is indicated as: ^#^, ∗*p* < 0.05; ^##^, ∗∗*p* < 0.01; ^###^, ∗∗∗*p* < 0.001.

LDH and CPK levels were significantly elevated in the DOX group (*p* < 0.001). Both MPH and HPH significantly reduced these levels toward control values (*p* < 0.001), whereas LPH did not produce a significant effect. Differences among honey groups correlated with their PAD-based quality grade (Fig. 4).

#### 3.3.4 Lipid profile

**Fig. 5:**
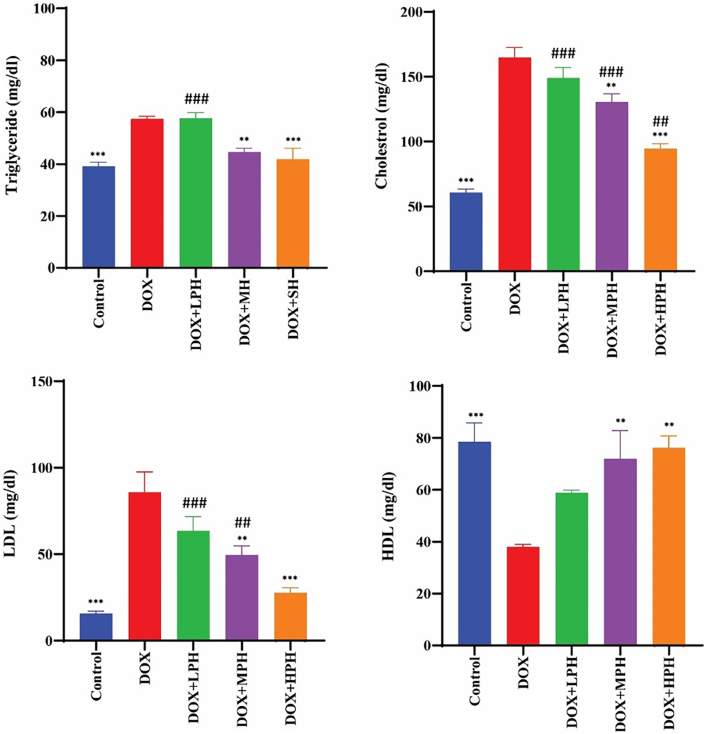
Effect of Honey on Serum Cholesterol, Triglycerides, LDL and HDL in DOX-treated Rats. All data are presented as mean ± SEM (mg/dL). ^#^Compare with Control, ∗ Compare with DOX, Statistical significance is indicated as: ^#^, ∗*p* < 0.05; ^##^, ∗∗*p* < 0.01; ^###^, ∗∗∗*p* < 0.001.

DOX caused significant disturbances in the lipid panel (*p* < 0.001). HPH treatment restored cholesterol, triglycerides, LDL, and HDL levels closest to control values, with no significant difference for most parameters, except for serum cholesterol, which remained slightly higher than control (*p* < 0.05). MPH and LPH also improved lipid profiles, but the effect was less pronounced, reflecting the hierarchical protective efficacy of honey quality (Fig. 5).

#### 3.3.5 Fasting blood glucose (FBS)

**Fig. 6:**
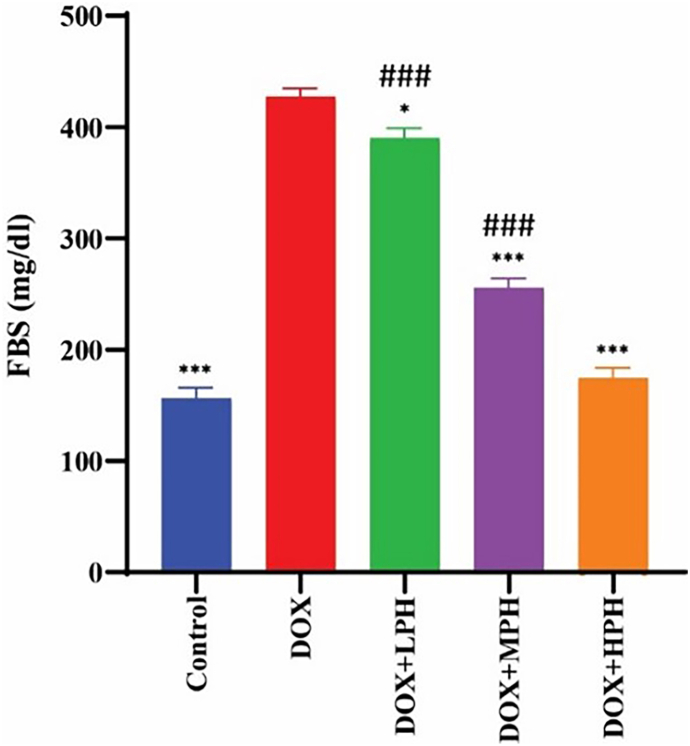
FBS variations between study groups. All data are shown as mean ± SEM and mg/dL. ^#^Compare with Control, ∗ Compare with DOX, Statistical significance is indicated as: ^#^, ∗*p* < 0.05; ^##^<∗∗*p* < 0.01; ^###^, ∗∗∗*p* < 0.001.

FBS levels were partially reduced by LPH (*p* < 0.05) but remained higher than control (*p* < 0.001). MPH and HPH treatments caused a highly significant reduction in FBS compared to the DOX group (*p* < 0.001), indicating dose-dependent glycemic protection (Fig. 6) (see Fig. 6).

### 3.4 Oxidative stress markers

DOX induced oxidative stress, as evidenced by increased MDA and decreased FRAP, DPPH, and Thiol levels. All honey treatments significantly decreased MDA levels, with HPH and MPH showing greater efficacy than LPH (*p* < 0.05–0.001). FRAP levels were significantly increased in all honey groups compared to DOX, with HPH surpassing control values (*p* < 0.001). DPPH scavenging activity was effectively restored in HPH and MPH groups, reaching levels comparable to control. Thiol levels were significantly elevated in MPH and HPH, with HPH showing the highest increase (*p* < 0.05), indicating enhanced antioxidant defence against DOX-induced toxicity (Table 3).

### 3.5 Histopathological findings of the liver

**Fig. 7:**
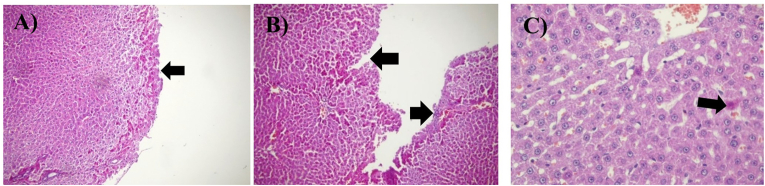
Microscopic images of liver tissue demonstrating eosinophilic cytoplasm within hepatocytes, captured at 100x and 200x magnification. The tissue sections were stained with hematoxylin and eosin (H&E). Black arrows indicate areas of cellular damage. (A) Dox-group, magnified 100×, (B) Another view of the Dox-group, magnified 100×, (C) HPH treated group, magnified 200×.

**Fig. 8:**
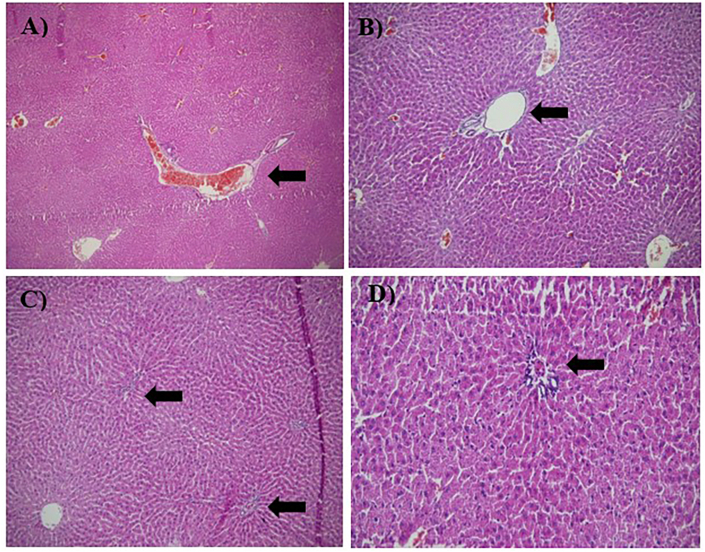
Microscopic images of liver tissue demonstrating Portal Vein Dilatation with Portal Hyperaemia captured at 100x and 200x magnification. The tissue sections were stained with hematoxylin and eosin (H&E). Black arrows indicate areas of cellular damage. (A) Dox-group, magnified 100×, (B) another view of the Dox-group, magnified 100×, (C) HPH-treated group, magnified 100×, (D) Represents the HPH-treated group, magnified 200×.

**Fig. 9:**
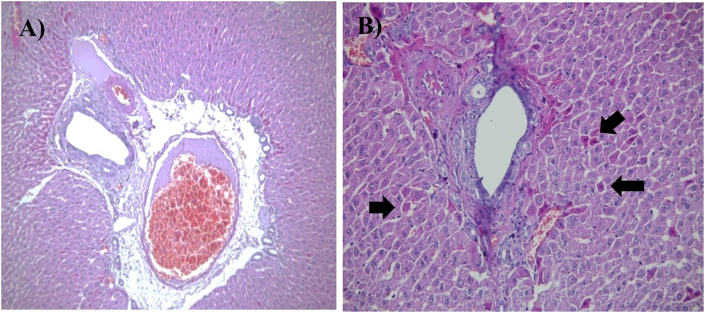
Microscopic images of liver tissue demonstrating Bile Ductular Proliferation with Hepatocellular Apoptotic Bodies at 200x magnification. The tissue sections were stained with hematoxylin and eosin (H&E). Black arrows indicate areas of cellular damage. (A) Represents the Dox-group, magnified 200×, (B) Another view of the Dox-group, magnified 200×.

DOX administration induced marked hepatic injury across all evaluated histopathological parameters compared with the control group, including severe sinusoidal dilation, portal and central vein congestion, portal area enlargement, bile duct proliferation, mononuclear inflammatory infiltration, hepatocyte degeneration, parenchymal necrosis, and periportal fibrosis. Pretreatment with honey produced varying degrees of protection. All three honey types significantly reduced sinusoidal dilation, hepatocyte degeneration, inflammatory cell infiltration, and hepatic necrosis relative to the DOX group (b∗∗∗), indicating a strong general hepatoprotective effect. MPH and HPH demonstrated superior efficacy by significantly improving portal vein congestion, portal area dilation, central vein congestion, and periportal fibrosis, whereas LPH honey showed only partial mitigation of these parameters. In the case of bile duct proliferation, only MPH and HPH exerted a significant protective effect (b∗∗∗), while LPH honey failed to produce measurable improvement. Overall, the histopathological data indicate that although all honey types ameliorated key features of DOX-induced hepatotoxicity, MPH and HPH honey provided the most consistent and robust protection across the majority of liver injury markers (Fig. 7, Fig. 8, Fig. 9).

Evaluation of the hepatic portal area further revealed that honey pretreatment significantly alleviated portal vein hyperaemia and dilatation relative to the DOX group, with significance levels indicated in Table 4.

**Table 4:** Histopathological changes in the liver among study groups.

Histopathological changes (Liver damage)	Control	DOX	DOX + LPH	DOX + MPH	DOX + HPH
Sinusoidal dilation	0.25 ± 0.27	3.47 ± 0.25 a∗∗∗	2.35 ± 0.20 a, ∗∗∗/b, ∗∗∗	1.35 ± 0.20 a, ∗∗∗/b, ∗∗∗	0.70 ± 0.31 a, ∗/b, ∗∗∗
Portal vein congestion	0.03 ± 0.08	2.37 ± 0.17 a∗∗∗	2 ± 0.14 a, ∗∗∗/b∗	1.25 ± 0.28 a, ∗∗∗/b, ∗∗∗	0.11 ± 0.20 b, ∗∗∗
Portal area dilation and enlargement	0.13 ± 0.19	2.63 ± 0.175 a∗∗∗	2.12 ± 0.16 a, ∗∗∗/b, ∗∗	1.40 ± 0.23 a, ∗∗∗/b, ∗∗∗	0.33 ± 0.25 b, ∗∗∗
Central vein congestion	0	2.73 ± 0.121 a, ∗∗∗	2.38 ± 0.19 a, ∗∗∗/b∗	1.33 ± 0.22 a, ∗∗∗/b, ∗∗∗	0.28 ± 0.24 b, ∗∗∗
Bile duct proliferation	0.25 ± 0.27	2.62 ± 0.22 a, ∗∗∗	2.38 ± 0.31 a, ∗∗∗	1.33 ± 0.25 a, ∗∗∗/b, ∗∗∗	0.25 ± 0.27 b, ∗∗∗
Pericentral inflammatory mononuclear cells	0.16 ± 0.25	2.77 ± 0.15 a, ∗∗∗	2.17 ± 0.25 a, ∗∗∗/b, ∗∗	1.15 ± 0.17 a, ∗∗∗/b, ∗∗∗	0.33 ± 0.40 b, ∗∗∗
Periportal mononuclear cells	0.250 ± 0.27	2.87 ± 0.33 a, ∗∗∗	2.10 ± 0.24 a, ∗∗∗/b, ∗∗∗	1.20 ± 0.23 a, ∗∗∗/b, ∗∗∗	1.00 ± 0.31 a, ∗∗/b, ∗∗∗
Hepatocyte degeneration (hepatocytes with pyknotic nuclei)	0.66 ± 0.25	3.33 ± 0.25 a, ∗∗∗	2.42 ± 0.20 a, ∗∗∗/b, ∗∗∗	1.42 ± 0.20 a, ∗∗∗/b∗∗∗	1.08 ± 0.20 a, ∗/b, ∗∗∗
Hepatic parenchymal necrosis	0	2.17 ± 0.25 a, ∗∗∗	1.25 ± 0.27 a, ∗∗∗/b, ∗∗∗	0.833 ± 0.25 a, ∗∗∗/b, ∗∗∗	0.66 ± 0.2 a, ∗∗∗/b, ∗∗∗
Periportal fibrosis	0	1 ± 0.31 a, ∗∗∗	0.16 ± 0.25 b, ∗∗∗	0.08 ± 0.20 b, ∗∗∗	0 b, ∗∗∗

Pathological parameters include sinusoidal dilation, portal vein congestion, portal area dilation and enlargement, central vein congestion, bile duct proliferation, pericentral and periportal mononuclear cell infiltration, hepatocyte degeneration (pyknotic nuclei), hepatic parenchymal necrosis, and periportal fibrosis. Values are presented as mean ± SD. a: significant difference compared to Control group, b: significant difference compared to DOX-induced hepatotoxicity group. Level of significance: ∗*p* ≤ 0.05; ∗∗*p* ≤ 0.01; ∗∗∗*p* ≤ 0.001. **Abbreviation:** DOX: doxorubicin; LPH: low PAD score honey; MPH: medium PAD score honey; HPH: high PAD score honey.

**Table 5:** Histopathological changes of kidney among study groups.

Histopathological changes (Kidney damage)	Control	DOX	DOX + LPH	DOX + MPH	DOX + HPH
Tubular epithelial cell degeneration	0.08 ± 0.20	2.80 ± 0.23 a∗∗∗	1.90 ± 0.20 a∗∗∗/b∗∗∗	1.62 ± 0.18 a∗∗∗/b∗∗∗	0.16 ± 0.25 b∗∗∗
Tubular atrophy	0.25 ± 0.27	3.07 ± 0.35 a∗∗∗	2.85 ± 0.23 a∗∗∗/b∗∗∗	2.00 ± 0.31 a∗∗∗/b∗∗∗	1.23 ± 0.29 a∗∗∗/b∗∗∗
Proteinaceous casts in tubules	0.08 ± 0.20	3.17 ± 0.25 a∗∗∗	2.90 ± 0.49 a∗∗∗	1.58 ± 0.21 a∗∗∗/b∗∗∗	0.16 ± 0.25 b∗∗∗
Glomerular hyperaemia	0.30 ± 0.24	2.23 ± 0.25 a∗∗∗	1.83 ± 0.20 a∗∗∗	1 ± 0.27 b∗∗∗	0.16 ± 0.25 b∗∗∗
Interstitial hyperaemia	0	2.37 ± 0.19 a∗∗∗	2.02 ± 0.23 a∗∗∗	1.32 ± 0.24 a∗∗∗/b∗∗∗	0.33 ± 0.25 b∗∗∗

Pathological parameters include tubular epithelial cell degeneration, tubular atrophy, proteinaceous casts in renal tubules, glomerular hyperaemia, and interstitial hyperaemia. Values are presented as mean ± SD. a: significant difference compared to Control group; b: significant difference compared to DOX-induced nephrotoxicity group. Level of significance: ∗*p* ≤ 0.05; ∗∗*p* ≤ 0.01; ∗***p* ≤ 0.001. Abbreviation:** DOX: doxorubicin; LPH: low PAD score honey; MPH: medium PAD score honey; HPH: high PAD score honey.

### 3.6 Histopathological findings of the kidney

**Fig. 10:**
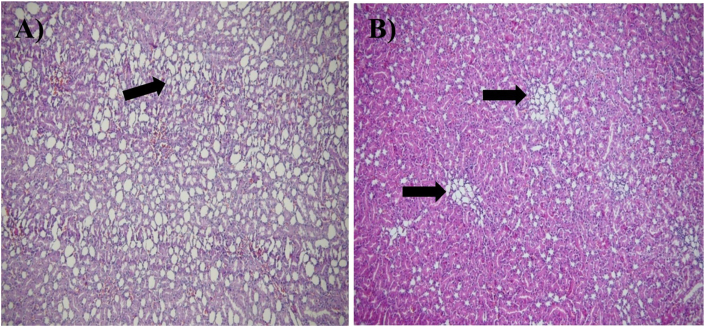
Microscopic images of kidney tissue demonstrating Renal Tubular Atrophy at 100x and 200x magnification. The tissue sections were stained with hematoxylin and eosin (H&E). Black arrows indicate areas of cellular damage. (A) Dox-group, magnified 100×, (B) MPH-treated group, magnified 100×.

**Fig. 11:**
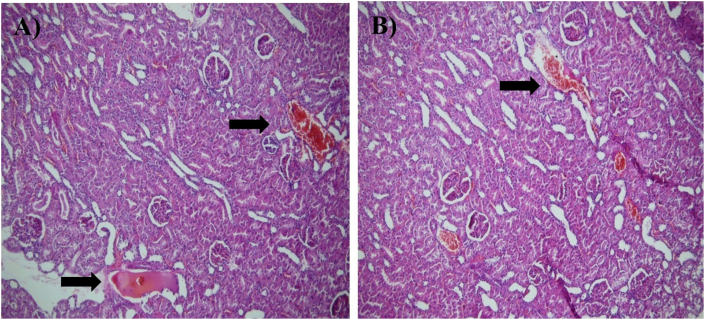
Microscopic images of liver tissue demonstrating Interstitial Hyperaemia with Tubular Casts at 100x magnification. The tissue s sections were stained with hematoxylin and eosin (H&E). Black arrows indicate areas of cellular damage. (A) Dox-group, magnified 100×, (B) Another view of the Dox-group, magnified 100.×.

DOX administration induced marked nephrotoxicity across all evaluated histopathological parameters compared with the control group, including tubular epithelial cell degeneration, tubular atrophy, proteinaceous casts in renal tubules, glomerular hyperaemia, and interstitial hyperaemia (see Table 5). Pretreatment with honey produced varying degrees of protection. All three honey types significantly ameliorated tubular epithelial cell degeneration relative to the DOX group (b, ∗∗∗), indicating a general cytoprotective effect. MPH and HPH demonstrated superior efficacy by significantly reducing tubular atrophy, proteinaceous casts, glomerular hyperaemia, and interstitial hyperaemia, whereas LPH honey showed only partial mitigation of these parameters. Notably, in the case of proteinaceous casts and glomerular hyperaemia, LPH honey failed to produce significant improvement (b, ∗∗∗). Overall, the histopathological data indicate that although all honey types mitigated key features of DOX-induced nephrotoxicity, MPH and HPH provided the most consistent and robust protection across the majority of kidney injury markers (Fig. 10, Fig. 11) [39].

## 4 Discussion

This study demonstrated that doxorubicin administration induced marked hepatorenal injury, metabolic disturbances, and oxidative stress, whereas honeys with higher PAD scores, characterized by greater phenolic content and antioxidant capacity, exerted stronger protective effects. The PAD classification effectively reflected the biochemical strength of each honey type, as higher-PAD honeys exhibited increased phenolic content, protein concentration, and antioxidant activity. These findings are consistent with previous reports indicating that the hepatoprotective effects of honey are closely associated with its phenolic composition and antioxidant potential [40,41].

DOX administration significantly increased serum markers of liver injury and fasting blood glucose, indicative of oxidative stress-induced hepatic damage and potential metabolic disturbances [42,43]. Pretreatment with MPH and HPH effectively restored these parameters to near-normal levels, whereas LPH honey exerted only partial protection. These findings are consistent with previous studies demonstrating honey's capacity to protect hepatocytes from oxidative stress and improve liver morphology, including reduction of ALT and AST levels, decreased hepatic malondialdehyde, and enhancement of antioxidant enzymes such as SOD and GPx [23,40,[44], [45], [46], [47]].

Histopathological analyses further supported these findings. Doxorubicin induced severe hepatic injury, including sinusoidal dilation, portal and central vein congestion, portal area enlargement, bile duct proliferation, mononuclear inflammatory infiltration, hepatocyte degeneration, parenchymal necrosis, and periportal fibrosis. Treatment with medium- and high-PAD-score honeys (MPH and HPH) significantly ameliorated most of these pathological features, whereas low-PAD-score honey (LPH) provided only partial protection. Disruption of calcium (Ca^2+^) homeostasis, driven by oxidative stress, inflammation, lipid peroxidation, and reduced membrane fluidity, may contribute to hepatocyte degeneration and parenchymal necrosis [39,48]. Bile duct proliferation, a response to localized inflammation, was reversed by MPH and HPH, restoring the hepatic sinusoidal structure to normal.

Similarly, DOX induced significant nephrotoxicity, as evidenced by elevated serum urea and creatinine, tubular epithelial degeneration, tubular atrophy, proteinaceous casts in the renal tubules, glomerular hyperaemia, and interstitial hyperaemia, indicating impaired kidney function. MPH and HPH significantly ameliorated these changes, while LPH honey provided limited protection. All three honey types, however, effectively reduced tubular epithelial degeneration, highlighting the inherent nephroprotective potential of honey. These nephroprotective effects of honey are consistent with previous studies demonstrating that honey feeding protects the kidney against cisplatin nephrotoxicity through suppression of inflammation, NFκB activation, and tubular epithelial cell death [25]. Additionally, phenolic-rich honey mitigated cisplatin-induced kidney injury by reducing oxidative stress, suppressing STAT3 and caspase-3, inhibiting pro-inflammatory mediators, and enhancing Nrf2 signaling [49]. In diabetic models, honey treatment significantly improved antioxidant enzyme activities (SOD, GR, CAT) and normalized serum urea and creatinine, further confirming its nephroprotective potential [50,51].

Protein accumulation in renal tubules, an early indicator of anticancer drug–induced nephrotoxicity, was attenuated in the honey-treated groups. The occurrence of necrotic features, including glomerular and interstitial hyperemia indicative of vascular damage associated with oxidative stress and systemic inflammation, was also reduced following honey treatment. These observations are consistent with previous studies reporting doxorubicin-induced apoptosis and necrosis in renal cells, as well as the protective effects of honey when combined with other natural products such as royal jelly and propolis [6,8].

Honey improved antioxidant defense–related parameters, as evidenced by enhancement of overall serum antioxidant capacity. Treatment with honey restored serum antioxidant markers, including DPPH scavenging activity, ferric reducing antioxidant power (FRAP), and total thiol content, while reducing malondialdehyde (MDA) levels, an index of lipid peroxidation. These changes indicate attenuation of oxidative stress following doxorubicin exposure [52]. These effects are attributed to honey's high content of phenolic compounds and flavonoids, which exhibit strong antioxidant activity [9,50].

In confirmation of all these results, it can be stated that honey, due to its extensive therapeutic properties, can be considered a medicinal agent. Its bioactive compounds, including flavonoids and polyphenols, contribute to its antioxidant, antibacterial, and anti-inflammatory capabilities. These properties render honey a valuable resource in the treatment of various diseases, from infections to chronic conditions [26,53]. Furthermore, honey is rich in phenolic compounds that exhibit potent antioxidant activity. Studies indicate that a higher phenolic content is associated with increased antioxidant capacity, which aids in neutralizing free radicals and reducing oxidative stress [54]. Additionally, specific phenolic acids, such as caffeic acid and p-coumaric acid, have been identified in various honey types, enhancing their health benefits [55]. Further studies are needed to explore the precise molecular pathways and clinical applicability of honey in chemotherapy-induced organ toxicity.

Although the findings demonstrated clear hepatoprotective and nephroprotective effects of PAD-classified honey against doxorubicin-induced toxicity, this study has several limitations. First, the experiments were conducted exclusively in male Wistar rats; therefore, potential sex-related differences were not evaluated. Second, the protective mechanisms were inferred from biochemical and histopathological findings rather than being directly confirmed at the molecular level. Finally, only a single dose and treatment duration were examined, which may not fully represent the broader therapeutic or protective range of honey.

## 5 Limitations

The present study has several limitations that should be acknowledged. First, the protective effects of PAD-scored honey were primarily inferred from biochemical, antioxidant, and histopathological findings, while molecular mechanisms were not directly investigated. In particular, oxidative stress–related enzymes such as superoxide dismutase (SOD) and glutathione peroxidase (GPx), as well as inflammatory signaling pathways including nuclear factor kappa B (NF-κB) and cytokine expression, were not assessed. Therefore, the mechanistic interpretations proposed in this study should be considered preliminary, and future investigations incorporating molecular and gene-expression analyses are warranted.

In addition, doxorubicin is known to induce cellular senescence in hepatic and renal tissues, which contributes to long-term organ dysfunction. However, specific markers of cellular senescence, such as senescence-associated β-galactosidase activity or the expression of p16^INK4a and p21, were not evaluated in the present study. Consequently, the potential effects of PAD-scored honey on doxorubicin-induced cellular senescence could not be determined and should be addressed in future studies.

Second, the PAD score was calculated based on single measurements of its biochemical components without technical replicates, which limits the assessment of analytical variability. Nevertheless, all assays used to construct the PAD score were selected based on widely accepted and up-to-date methods for honey quality and bioactivity assessment and were performed according to standardized AOAC/ISO protocols under identical laboratory conditions to minimize measurement bias.

Third, the relatively small sample size (n = 7 rats per group) may limit the statistical power to detect subtle or moderate differences among groups. Although post hoc power analysis indicated adequate power to detect large effect sizes, smaller effects may have remained undetected.

Finally, body weight changes and food intake were not systematically monitored during the intervention period, as the primary objective of the study was to evaluate protection against doxorubicin-induced hepatorenal injury rather than metabolic outcomes. Although fasting blood glucose was measured, this parameter alone may not fully capture the metabolic effects of honey consumption. Therefore, future studies should include a honey-only control group along with comprehensive metabolic monitoring to better clarify the effects of medicinal honey on glucose homeostasis and energy balance.

## 6 Conclusion

The present study demonstrates that honey classified according to the PAD scoring system, particularly medium- and high-PAD honeys, exerts protective effects against doxorubicin-induced hepatic and renal injury in a rat model. These effects were reflected by improvements in serum biochemical indices, modulation of oxidative stress–related parameters, and attenuation of histopathological damage. The higher phenolic content and antioxidant capacity of medium- and high-PAD honeys may contribute to their enhanced protective effects against doxorubicin-induced tissue injury. While low-PAD honey showed limited efficacy, medium- and high-PAD honeys consistently exhibited greater protective capacity in this experimental setting. Overall, these findings support the relevance of PAD-based honey classification for evaluating bioactive properties and suggest a potential role for phenolic-rich honey as a protective nutritional factor in preclinical models of chemotherapy-induced organ toxicity. However, further mechanistic investigations and carefully designed studies are required before any clinical implications can be considered.

## CRediT authorship contribution statement

**Mahdi Honarbakhsh:** Writing – original draft, Visualization, Project administration, Methodology, Conceptualization. **Nafiseh Erfanian:** Writing – original draft, Validation, Methodology, Formal analysis. **Amir Hossein Saberi:** Resources, Investigation, Data curation. **Pouria Mohammad Parast Tabas:** Visualization, Resources, Data curation. **Motahhareh Mohammadi:** Visualization, Resources, Methodology. **Ahmad Bavali-Gazik:** Writing – original draft, Formal analysis, Data curation. **Asghar Zarban:** Writing – original draft, Validation, Supervision, Project administration, Methodology.

## Data statement

The datasets generated and analysed during the current study are not publicly available due to restrictions related to data ownership and participant confidentiality but are available from the corresponding author on reasonable request.

## Funding

This research did not receive any specific grant from funding agencies in the public, commercial, or not-for-profit sectors.

## Glossary

PAD: Phenolic Content and Protein, Antioxidant Capacity and Diastase Activity
AKI (acute kidney injury): Sudden decline in kidney function causing impaired filtration and accumulation of nitrogenous wastes
ALP (alkaline phosphatase): Liver/bone enzyme; elevated serum levels can indicate cholestasis or hepatobiliary injury
ALT (alanine aminotransferase): Hepatocellular enzyme; serum elevation reflects liver cell injury
ANOVA: One-way analysis of variance; compares means across multiple groups assuming normality
Antioxidant capacity: Overall ability of a sample to neutralize reactive oxygen/nitrogen species
AST (aspartate aminotransferase): Enzyme released with liver or muscle injury; often interpreted with ALT
Banff classification: Standardized scoring system for renal histopathology, widely used in kidney pathology
Bradford assay: Colorimetric method to quantify protein concentration using Coomassie dye binding
Cardiac enzymes (CPK, LDH): cardiac or tissue injury; elevated after cytotoxic insult
CKD (chronic kidney disease): Long-term loss of renal function, often following unresolved acute injury
CPK (creatine phosphokinase): Enzyme released from muscle; increases with myocyte damage
Diastase activity: Amylase activity in honey; indicates freshness/enzymatic integrity
DOX (doxorubicin): Anthracycline chemotherapeutic; inhibits topoisomerase II and induces oxidative toxicity
DPPH assay: Free-radical scavenging test; higher quenching indicates stronger antioxidant activity
Ellman assay (thiol): Quantifies total sulfhydryl groups using DTNB reagent as a proxy for antioxidant status
FBS (fasting blood glucose): Serum glucose after fasting; reflects glycaemic status
FCR (Folin–Ciocalteu reagent): Reagent to estimate total phenolic content via colorimetric response
FRAP (ferric reducing antioxidant power): Assay measuring reduction of Fe^3+^ to Fe^2+^; indicates antioxidant power
GAE: Gallic acid equivalents; unit to express total phenolic content
Glomerular/interstitial hyperaemia: Increased blood content in glomeruli or interstitium indicating vascular congestion
H&E (hematoxylin & eosin): Routine histological stain for cellular and tissue morphology
Hepatorenal toxicit: Combined liver and kidney injury induced by a toxic agent
Hepatocyte degeneration/pyknos: Structural deterioration of liver cells; pyknotic nuclei indicate advanced injury
HPH/MPH/LP: High/Medium/Low PAD score honey categories reflecting graded biochemical quality
Histopathological activity index (Knodell): Composite score grading severity of hepatic lesions
Intraperitoneal(i.p.): Route of administration by injection into the peritoneal cavity
LDH (lactate dehydrogenase): Cytosolic enzyme; rises with tissue damage and haemolysis
Lipid peroxidation: Oxidative degradation of lipids producing aldehydes such as MDA
MDA (malondialdehyde): End-product of lipid peroxidation; marker of oxidative stress
